# Conservation of artists' acrylic emulsion paints: XPS, NEXAFS and ATR-FTIR studies of wet cleaning methods†

**DOI:** 10.1002/sia.5376

**Published:** 2014-02-17

**Authors:** EA Willneff, BA Ormsby, JS Stevens, C Jaye, DA Fischer, SLM Schroeder

**Affiliations:** a School of Chemical Engineering and Analytical Science, The University of ManchesterThe Mill, Sackville Street, Manchester, M13 9PL, UK; b TateMillbank, London, SW1P 4RG, UK; c School of Chemistry, The University of ManchesterBrunswick Street, Manchester, M13 9PL, UK; d National Institute of Standards and TechnologyGaithersburg, MD, 20899, USA

**Keywords:** acrylic emulsion paint, Heritage Science, XPS, NEXAFS, ATR-FTIR, microemulsion, cleaning, residue, pigment

## Abstract

Works of art prepared with acrylic emulsion paints became commercially available in the 1960s. It is increasingly necessary to undertake and optimise cleaning and preventative conservation treatments to ensure their longevity. Model artists' acrylic paint films covered with artificial soiling were thus prepared on a canvas support and exposed to a variety of wet cleaning treatments based on aqueous or hydrocarbon solvent systems. This included some with additives such as chelating agents and/or surfactants, and microemulsion systems made specifically for conservation practice. The impact of cleaning (soiling removal) on the paint film surface was examined visually and correlated with results of attenuated total reflection Fourier transform infrared, XPS and near-edge X-ray absorption fine structure analyses – three spectroscopic techniques with increasing surface sensitivity ranging from approximately − 1000, 10 and 5 nm, respectively. Visual analysis established the relative cleaning efficacy of the wet cleaning treatments in line with previous results. X-ray spectroscopy analysis provided significant additional findings, including evidence for (i) surfactant extraction following aqueous swabbing, (ii) modifications to pigment following cleaning and (iii) cleaning system residues. © 2014 The Authors. *Surface and Interface Analysis* published by John Wiley & Sons, Ltd.

## Introduction

There is an increasing need for fundamental research informing the conservation and restoration of 20th century acrylic paintings. In contrast to oil paintings, for which conservator-restorers can draw on an extensive body of detailed previous research, there is much less extant knowledge that permits informed choices of particular cleaning strategies for works of art made from acrylic emulsion paints. To address this knowledge gap, the impact of wet cleaning agents on the bulk film and surface properties of artists' acrylic paint films has been investigated over the last decade^1^–^3^ by using several types of Fourier transform infrared (FTIR) spectroscopies^1^,^2^ alongside mass spectrometry,^4^ AFM,^3^ SEM-EDX analysis and visual inspection. Also relevant for the present study is recent work on acrylic glass, for example, by atomic force microscopy.^5^ While all of these techniques probe the surface of the paint film, information on the chemistry of the crucial uppermost surface (<10 nm) is nonexistent. In light of this, the analytical value of XPS, which has not been applied in this context before, in combination with near-edge X-ray absorption fine structure (NEXAFS) has been explored in the present work by characterising azo yellow (PY3) artists' acrylic emulsion paint films from two manufacturers before and after wet cleaning treatments. The results were examined in conjunction with those from attenuated total reflection-FTIR (ATR-FTIR) spectroscopy and visual inspection, which are routinely used to assess the surfaces of these paint films. A selection of these results is presented here to highlight the benefit of applying surface X-ray spectroscopy surface analysis in improving our understanding of the impact of wet cleaning treatments on artists' acrylic emulsion paint films.

## Experimental

### Artists' acrylic paint films

Acrylic emulsion paint films were prepared on triple primed 100% cotton duck canvas (Russel and Chapel, London). Artists paints, Golden Heavy Body Acrylics Hansa Yellow Light and Talens Rembrandt Azo Yellow Lemon, both containing PY3 azo yellow organic synthetic pigment, were applied to the canvas support using a draw-down technique on a Sheen Instruments film caster, to a wet thickness of approximately 800 µm and dry thickness of 200–250 µm, as measured with a digital calliper. The resin for the Golden paint was a p*n*(butyl acrylate/methyl methacrylate) [p*n*(BA/MMA)] copolymer while the resins for the Talens paint was a p(ethyl acrylate/methyl methacrylate) [p(EA/MMA)] copolymer with detectable amounts of a chalk (CaCO_3_) extender. ATR-FTIR spectra indicated that the surfactant used as a pigment dispersant in the bulk paint formulation had not migrated to the surface of either dry films. Because migrated surfactant is suggested to play a role in soiling adhesion and pigment mobility and known to change macroscopic properties of the paint film such as gloss,^6^ knowledge of its presence or absence may be relevant to interpreting the relationship between surface chemistry and cleaning efficacy. Films were divided into squares (∽1 cm^2^) and cleaned with one of 25 cleaning agents in each square. Four of these areas were selected for spectroscopic analysis and details of their cleaning treatment presented below.

### Artificial soiling

To simulate the effect of many years of passive soiling, the model films were allowed to dry in ambient conditions for 5 months before brushing on an artificial soiling mixture^7^ approximating typical indoor particulate soiling. Before cleaning treatments, the soiling was allowed to dry for 2 weeks on the Golden paint film, while the Talens film underwent cleaning studies after 2 days. Although longer drying is ideal, access arrangements to experimental techniques made this impossible.

### Wet cleaning agents

The water (W) used was deionized (DI) (Purite, D700 deionizer). A 100% aliphatic petroleum spirit (PS) (VWR International) with a boiling point of 120–160 °C was used as received. ‘Ecosurf + triammonium citrate (TAC)’ (ET) consisted of a solution of 1% v/v ECOSURF™ EH-9 (The Dow Chemical Company) and 1% w/v TAC chelating agent in deionized water. The microemulsion (ME) was a water-in-oil microemulsion comprised of proportions of lauryl ammonium sulphate (LAS), low-molecular-weight-alcohol-based cosolvents, a Shellsol D38 mineral spirits solvent continuous phase and deionized water.^3^

### Cleaning treatment simulation

Each cleaning agent was applied to an approximately 1 cm^2^ square area of the paint film by dipping a pre-rolled cotton swab on a wooden applicator (Puritan) into the solution and then rolling the swab back and forth (one roll) across the paint film to a total of 20 rolls and dried in ambient conditions. In standard conservation practice, the cleaning step would have been immediately followed by a clearance step, i.e. swabbing of the cleaned area with a liquid likely removes any cleaning residues. This was avoided in these preliminary studies so possible residues from the cleaning agents could be identified but will be addressed in subsequent publications.

Samples for spectroscopic analysis were prepared using a single-hole punch, resulting in circular discs with a diameter of 6 mm.

### Fourier Transform Infrared Spectroscopy

Attenuated total reflection-FTIR spectra were collected on a Nicolet iS10 system (Thermo Scientific) with a Smart Omni ATR sampler containing a single-bounce germanium crystal. The penetration depth of the beam is 0.66 µm at 2000 cm^−1^. Background and sample spectra were acquired for each sample on two to three different spots in 64 scans with a resolution of 4 cm^−1^. Averages of these spectra are presented here. The cleanest part of a sample (judged visually) was analysed to explore the surface chemistry of the most effectively cleaned area.

### X-ray photoelectron spectroscopy

X-ray photoelectron spectra were collected on a Kratos Axis Ultra spectrometer operating with (i) a monochromatic Al K_α_ X-ray anode (1486.69 eV) at 180 W (15 kV, 12 mA), (ii) a hemispherical analyser in electrostatic mode (*p* < 10^−7^ mbar) and (iii) charge neutralisation. Survey XP spectra were acquired in a single sweep with a pass energy of 80 eV, in steps of 0.35 eV and dwell time of 150 ms, giving collection times of approximately 9 min per spectrum. High-resolution XP spectra were acquired in a single sweep with a pass energy of 20 eV, in steps of 0.1 eV with a dwell time of 200 ms, giving a collection time of 1 min per spectrum. The circular punch outs from the paint film were mounted on the sample bar with double-sided tape. As with FTIR, the visually cleanest part of each sample was analysed, by aligning in the focal point of the electron analyser. Data analysis was carried out with CasaXPS. Binding energies were referenced to a primary hydrocarbon peak at set to 285.0 eV, which required a correction of approximately +3–3.5 eV to the experimental data. XP spectra acquired over 0–200 eV binding energy (BE) of elements at low concentration were noisy and smoothed for better clarity.

### Near-edge X-ray absorption fine structure

Near-edge X-ray absorption fine structure measurements were performed at the U7a beamline of the National Synchrotron Light Source at Brookhaven National Laboratory, NY.^7^,^8^ Partial electron yield spectra for the Ca L-edge were collected via a channeltron electron multiplier and with an entrance grid bias (EGB) of −50 and −200 V.

## Results and discussion

### Visual inspection

Macroscopic effects of cleaning treatments on the paint films were initially assessed by eye. The observations confirmed previous reports of paint brand (formulation) dependency of these effects.^3^ The relative cleaning efficacies of the systems were immediately apparent. Soiled paint areas swabbed with effective cleaning systems were very similar in colour to the yellow as-prepared unsoiled areas while those soiled areas swabbed with less effective cleaning systems remained partially grey from residual soiling. On the basis of these criteria, the cleaning systems were ranked as follows, from least to most effective: PS ≤ DI water (W) < Ecosurf + TAC (ET) = microemulsion (ME). Furthermore, it was also apparent from the transfer of yellow pigment to the cotton swab that more PY3 pigment was removed from the Talens films by the cleaning action than from the Golden films.

### Fourier Transform Infrared Spectroscopy

**Figure 1:**
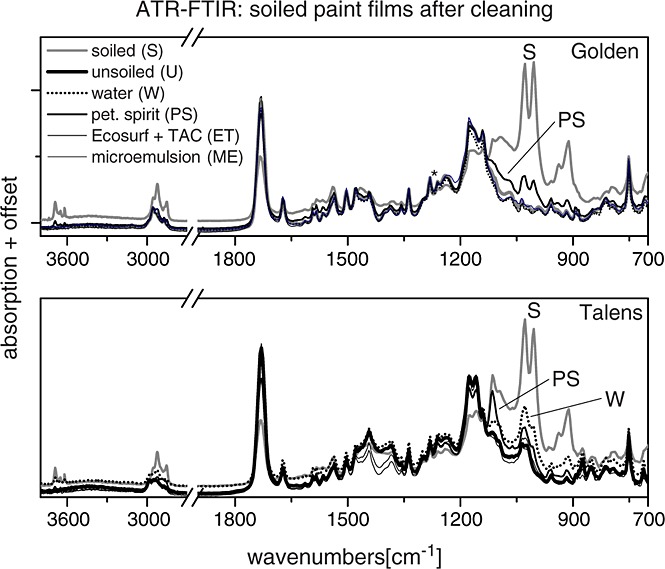
Attenuated total reflection Fourier transform infrared (ATR-FTIR) spectra of paint films support trends in cleaning efficacy as identified by visual inspection.

The ATR-FTIR (Fig. 1) readily identified surface soiling from a group of bands between 3600 and 3700 cm^−1^ and several bands in the fingerprint region at ca 1030 and 1000 cm^−1^ associated with vibrations of aluminosilicate materials in the soiling mixture, e.g. lattice water and Si–O stretches respectively. Further, bands associated with residues from the hydrocarbon makeup solvent of the soiling mixture were observed at ca 2920 and 2950 cm^−1^ (C–H stretch). The spectra of the soiled paint films after cleaning (Fig. 1) confirmed the trends in cleaning efficacy observed by eye. The vibrational bands associated with surface soiling diminished in proportion to cleaning efficacy of the surface treatment. Whereas soiling bands were completely gone after cleaning with the ME and ET, some soiling was still visible after cleaning with PS on both films and also water on the Talens film. Other modifications to components of the paint film such as surfactant, which was not detected on either paint films before or after cleaning, or pigment were not observed.

**Figure 2:**
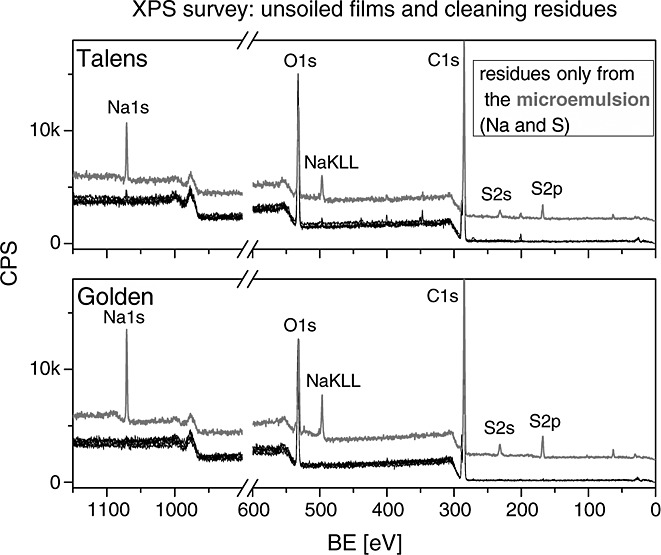
The primary elemental composition of the unsoiled paint films before and after cleaning is visible in the survey XP spectra. Na and S lines are associated with microemulsion residues.

### X-ray photoelectron spectroscopy

More in-depth analysis of the paint film surfaces by X-ray spectroscopy confirmed the trends in relative cleaning efficacy. However, this analysis also revealed the presence of cleaning residues (without a clearance step) in addition to other subtle changes to the paint film surface, some of which are highlighted further in the succeeding text.

The survey XP spectra (of the unsoiled paint films) are dominated by C and O, which originate from the acrylic binder (Fig. 2). Additionally, strong Na and S emissions are visible on the sample cleaned with the microemulsion. These residues stem from the anionic LAS surfactant in the cleaning agent. The presence of LAS residues was evident also from subtle changes in the C 1s and O 1s XP spectra (not shown here). Identification of the LAS residues was not possible by visual inspection or ATR-FTIR. Work is underway to determine how these residues are affected by a clearance step, i.e. a final cleaning step that is standard conservation practice aimed at removing cleaning system residues usually carried out with deionised water or mineral spirits as well as how to identify residues (if any) from similar microemulsions without LAS or other components with inorganic functional groups.

**Figure 3:**
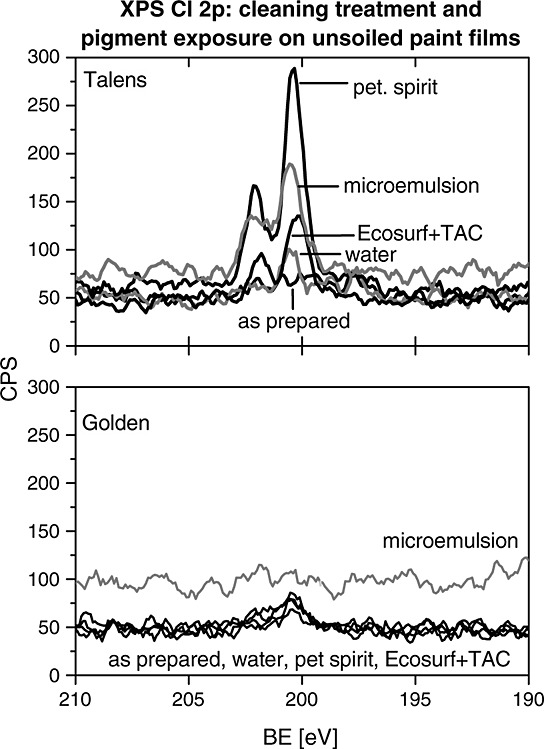
Cl 2p XP spectra of the Talens yellow paint film are very responsive to cleaning treatments.

During cleaning, pigment transfer onto the cotton swabs was repeatedly observed during cleaning of the Talens film but to varying degrees depending on the cleaning agent used. While ATR-FTIR did not detect associated modifications to the surface of the paint film, XP spectra of chlorine – an element of the azo yellow pigment and primary contributor to the chlorine signal in these measurements – proved sensitive (Fig. 3). In the Cl 2p emission from the Talens films, the Cl signal was more intense after cleaning with solvent-based systems (PS and ME) than after cleaning with water-based agents (W and ET).

In contrast, the Cl 2p signal, after the same cleaning treatments on the corresponding Golden paint film, was virtually indistinguishable from the baseline before and after cleaning treatments. These manufacturer-dependent and treatment-dependent changes in the Cl 2p XP signal associated most likely with the azo yellow pigment could be explained by greater pigment mobility of unbound or poorly bound pigment in the Talens paints, perhaps mediated by increased solubility in the hydrocarbon solvents. The lack of similar trends in the Cl 2p XP spectra on the Golden film suggests that the pigment in the Golden formulation is more resistant towards the particular chemistry of the wet cleaning agents, possibly because the film is more medium rich at the film surface, i.e. covered by acrylic resin.

In comparison with Cl 2p XP spectra of the unsoiled cleaned Talens paint film, the Cl 2p XP spectra of the soiled film, after cleaning treatments, was much weaker in intensity and hardly distinguishable from the background, suggesting that while some pigment is transferred during cleaning to the swab rolls, the soiling layer appears to have provided some protection to the underlying paint film. This supports the standard conservation practice of only cleaning until an area appears visually clean, i.e. until the soiling layer has been sufficiently reduced.

**Figure 4:**
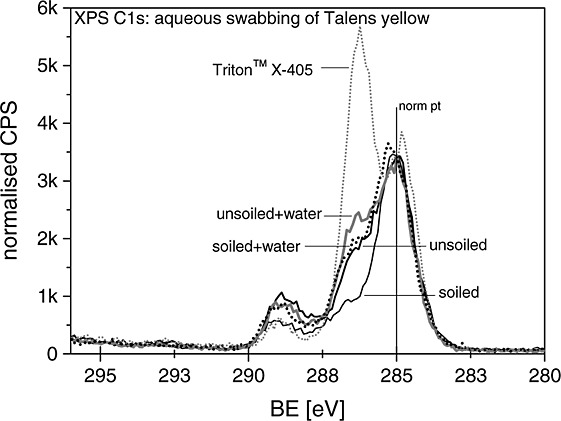
The C 1s XP spectrum of the unsoiled Talens paint film after aqueous swabbing has a more intense shoulder at +1.5 eV associated with C–O moieties likely to be associated with the ethoxylate side chain of the Triton-X-405-type surfactant extracted during this cleaning treatment.

Evidence for surfactant at the surface of the unsoiled Talens yellow paint was not identified by any technique before cleaning. However, in the C 1s XP spectrum (Fig. 4) of the unsoiled Talens yellow paint film, a slight increase in the C–O ether component at +1.5 eV (relative to the primary C–H XP component at 285 eV) was observed after aqueous swabbing. The type of surfactant in these paints is non-ionic, of the Triton type and hydrophilic, making it amenable to aqueous extraction from the bulk paint film. Talens paints in general have previously been observed to be more surfactant rich than Golden paints.^8^,^9^ The subtle increase in the C–O ether component of the C 1s XP spectrum suggests that the concentration of this PEO Triton-type surfactant has increased slightly by aqueous swabbing. This change was not observed on the soiled Talens yellow film, suggesting that surfactant in the underlying paint film was unaffected by the cleaning treatment. This is reminiscent of the apparent protective function of the applied soiling layer in preventing pigment disruption at the paint film discussed previously in the context of the Cl 2p XP spectra.

### Near-edge X-ray absorption fine structure

**Figure 5:**
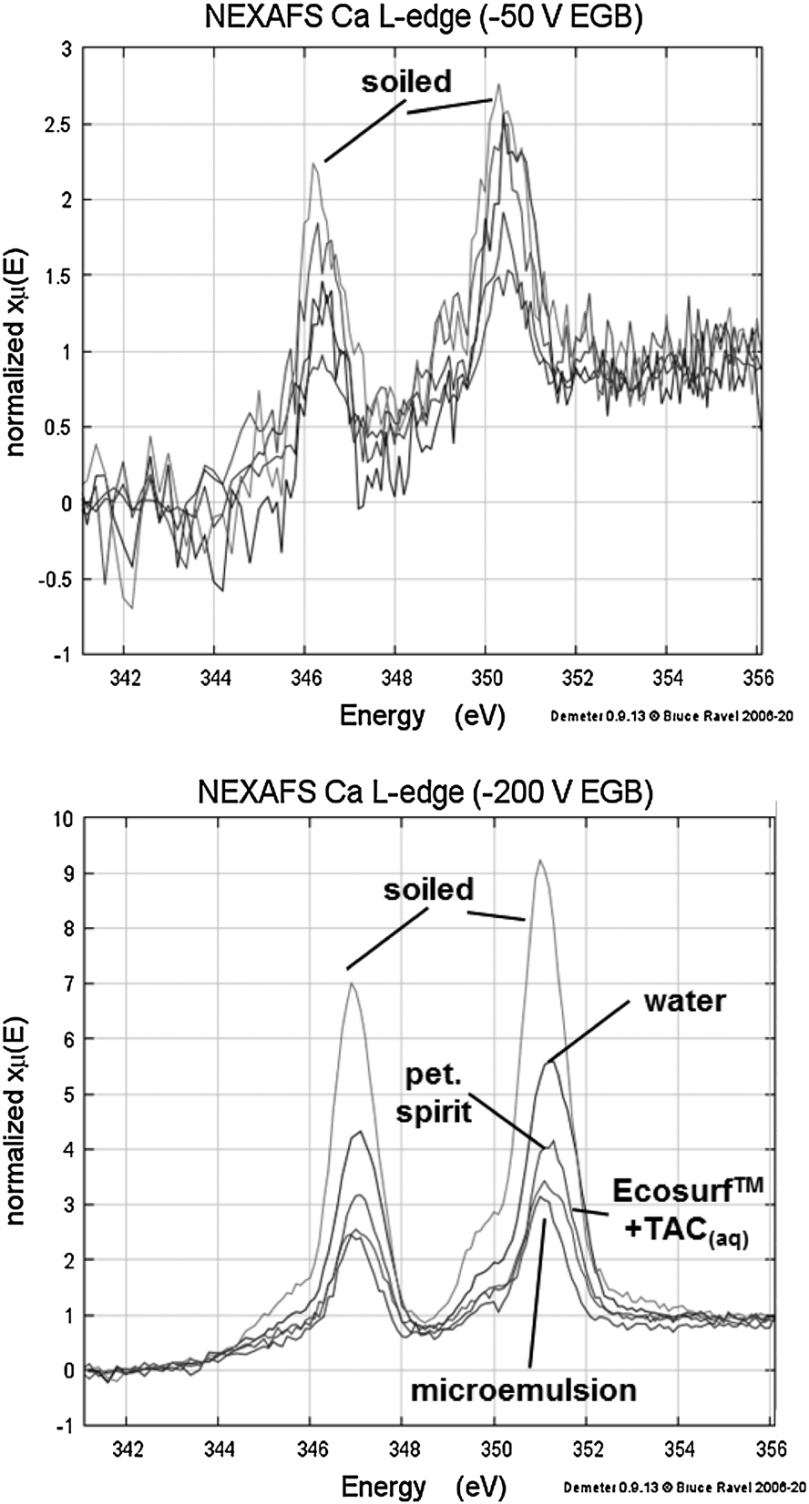
Calcium L-edge near-edge X-ray absorption fine structure (NEXAFS) spectra suggest stratification of calcium in the uppermost paint film surface with the most surface-sensitive spectra (bottom) very responsive to cleaning treatments.

Near-edge X-ray absorption fine structure provided additional insight. One is evidence for the stratification of paint film components in the uppermost surface, which was obtained by variation of the surface sensitivity through the use of a biased grid at the detector entrance.^9^,^10^ For example, Ca L-edge NEXAFS spectra (Fig. 5) of soiled Talens yellow films were acquired at two different EGBs. Applying an EGB of −50 V results in a higher signal sampling depth than for an EGB of −200 V. The Ca L-edge signal is much less intense in the more bulk-sensitive spectra taken with an EGB of −50 V than in the more surface-sensitive spectra taken with an EGB of −200 V. This indicates stratification in the uppermost surface of the paint film of calcium originating from the applied soiling layer and/or chalk extender present in the bulk paint. Moreover, data acquired with an EGB of −50 V were virtually unaffected by cleaning treatments, while the surface-sensitive spectra acquired at an EGB of −200 V were very responsive to cleaning treatments, indicating a decrease in Ca L-edge intensity broadly in proportion to the visually observed cleaning efficacy of the surface treatment.

## Conclusions

Two yellow (PY3) paint films prepared from artists' acrylic emulsion paint from different manufacturers were exposed to four cleaning treatments. Films were assessed by eye for the impact of cleaning and results correlated with those of ATR-FTIR, XPS and NEXAFS analyses. All techniques established the relative cleaning efficacy of the wet cleaning treatments in order from least to most effective to be: PS ≤ DI water (W) < Ecosurf + TAC (ET) = microemulsion (ME) – in line with previous results. However, significant additional findings arose from the X-ray spectroscopic analysis, including (i) indications of surfactant extraction following aqueous swabbing, (ii) modifications to pigment at the paint film surface and (iii) the identification of cleaning residues. These subtle modifications at the very surface of the paint film may have consequences for the preservation and appearance of works of art made with these paints. The potential application of NEXAFS as a ‘depth profiling’ tool for these materials should also be examined further. Currently, more systematic investigations on these paint films are being carried out employing a wider variety of surface treatments (e.g. ageing and other microemulsions) and paint materials (e.g. paint films from different manufacturers with different pigments). This information will inform ongoing research into the most appropriate, minimal-risk way to conserve and preserve this growing proportion of modern and contemporary works of art.
